# Custo-efetividade do Stent Farmacológico na Intervenção Coronariana Percutânea no SUS

**DOI:** 10.36660/abc.20180292

**Published:** 2020-07-28

**Authors:** João Addison Pessoa, Esmeralci Ferreira, Denizar Viana Araújo, Edirley Maia, Felipe Souza Maia da Silva, Maurício Salles de Oliveira, Denilson Campos de Albuquerque

**Affiliations:** 1 Universidade do Estado do Rio de Janeiro Rio de JaneiroRJ Brasil Universidade do Estado do Rio de Janeiro, Rio de Janeiro, RJ - Brasil; 2 Hospital São Lucas Nova FriburgoRJ Brasil Hospital São Lucas, Nova Friburgo, RJ - Brasil

**Keywords:** Infarto do Miocárdio, Intervenção Coronária Percutânea, Stents Farmacológicos, Reestenose Coronária, Análise de Custo e Benefício, Sistema Único de Saúde (SUS

## Abstract

**Fundamento:**

O uso do stent farmacológico (SF) comparado ao stent não farmacológico (SNF) na intervenção coronariana percutânea (ICP) reduziu o percentual de reestenose, porém sem impacto na mortalidade, com aumento no custo. A literatura carece de estudos randomizados que comparem economicamente esses dois grupos de stents na realidade do Sistema Único de Saúde (SUS).

**Objetivo:**

Estimar a razão custo-efetividade incremental (RCEI) entre SF e SNF na coronariopatia uniarterial em pacientes do SUS

**Métodos:**

Pacientes com coronariopatia uniarterial sintomática foram randomizados em 3 anos para uso de SF ou SNF durante a ICP, na proporção de 1:2, com seguimento clínico de 12 meses. Foram avaliados reestenose intrastent (RIS), revascularização da lesão-alvo (RLA), eventos adversos maiores e custo-efetividade (CE) de cada grupo. Os valores de p < 0,05 foram considerados significativos.

**Resultados:**

No grupo SF, dos 74 pacientes (96,1%) que completaram o acompanhamento, ocorreu RIS em 1(1,4%), RLA em 1 (1,4%), óbito em 1 (1,4%), sem trombose. No grupo SNF, dos 141 pacientes (91,5%),ocorreu RIS em 14 (10,1%), RLA em 10 (7,3%), óbito em 3 (2,1%) e trombose em 1 (0,74%). Na análise econômica, o custo do procedimento foi de R$ 5.722,21 no grupo SF e de R$4.085,21 no grupo SNF. A diferença de efetividade a favor do grupo SF por RIS e RLA foi 8,7% e 5,9%, respectivamente, com RCEI de R$ 18.816,09 e R$ 27.745,76.

**Conclusões:**

No SUS, o SF foi custo-efetivo, em concordância com o limiar de CE preconizado pela Organização Mundial da Saúde. (Arq Bras Cardiol. 2020; 115(1):80-89)

## Introdução

Dados extraídos da Pesquisa Nacional de Saúde^1^ (PNS), de 2013, estimaram que 72,1% da população utilizaria o Sistema Único de Saúde (SUS) para tratamento médico ou odontológico. Segundo o levantamento do número de óbitos no Brasil por grupo de causas entre 2004 e 2014, estima-se em 1.069.653 (8,8%) o número de indivíduos que morreram por infarto agudo do miocárdio (IAM) ou outras doenças isquêmicas do coração. Nesse sentido, é importante a elaboração de medidas sustentáveis de prevenção e tratamento dessa enfermidade no SUS.^2^

No Brasil, os primeiros stents farmacológicos (SF, recobertos com sirolimus e paclitaxel) ficaram restritos ao sistema suplementar de saúde pelo alto custo. Estudos iniciais, tanto no Brasil como no exterior, não demostraram custo-efetividade (CE) para o implante em todos os casos, sugerindo seu uso em situações de maior risco para reestenose.^3 - 6^

As limitações descritas impuseram o desenvolvimento de novos SF, denominados segunda geração. Esses, com novos fármacos antiproliferativos e melhora na plataforma com hastes metálicas mais finas (ligas cromo-cobalto, platina-cobalto) proporcionaram melhor aposição do stent e menor superfície para endotelizacão. Polímeros biocompatíveis diminuíram o processo inflamatório local, reduzindo os casos de trombose tardia.^7 , 8^

Apesar de mais de uma década após o início de sua comercialização, o uso dos SF no SUS continuou limitado, mesmo com menor valor monetário e resultados mais favoráveis.

Em 2014, a Comissão Técnica de Incorporação de Tecnologias^9^ (CONITEC) reconheceu o custo-benefício do implante do SF em pacientes diabéticos, com vasos de pequeno calibre (< 2,5 mm) e com lesões longas (> 18 mm). Apesar de o preço de mercado do SF ser superior ao do stent não farmacológico (SNF), o valor sugerido no relatório (R$ 2.034,50/código 070204061-4) foi idêntico para as duas próteses, inviabilizando naquele período seu uso sistemático em serviços privados conveniados com o Ministério da Saúde.

Segundo dados do DATASUS (TABNET),^10^ no ano de 2008, foram realizadas 44.138 angioplastias coronarianas com ou sem stent. Oito anos após, foram realizadas 79.997 angioplastias. Com esse aumento expressivo de procedimentos (72,84%), pode-se projetar um aumento de casos de reestenose que, potencialmente, poderiam ser reduzidos com o uso mais liberal do SF no SUS.

Apesar do cenário favorável para a plena incorporação do SF ao SUS, ela necessita de evidência científica baseada na realidade brasileira. Sendo assim, este trabalho tem por objetivo analisar e estimar a razão custo-efetividade incremental (RCEI) entre SF e SNF em pacientes do SUS.

## Objetivos

Avaliar a CE e os eventos adversos maiores do uso do SF comparado ao SNF em pacientes uniarteriais submetidos à intervenção coronariana percutânea (ICP).

## Métodos

Estudo clínico, randomizado, realizado com pacientes indicados para ICP, no período de novembro de 2013 a outubro de 2016, atendidos no Setor de Hemodinâmica do Hospital Universitário Pedro Ernesto (HUPE/UERJ) e do Hospital São Lucas de Nova Friburgo (Nova Friburgo, RJ). O estudo foi aprovado pelo Comitê de Ética em Pesquisa das instituições sob o nº 923660. Todos os pacientes assinaram o Termo de Consentimento Livre e Esclarecido (TCLE), de acordo com a Resolução CNS nº 466 de 12/12/12.

Foram estudados 231 pacientes, de ambos os sexos, com lesões uniarteriais e indicação de ICP, após realização prévia de cineangiocoronariografia, com sintomas de angina ou com exames não invasivos comprovando isquemia miocárdica. Foram adotados os seguintes critérios de inclusão: (1) pacientes com idade ≥ 18 anos; (2) lesões angiograficamente significativas (> 70%) em uma coronária de grande importância anatômica, com irrigação de grande área de músculo cardíaco, relacionada à presença de isquemia ou sintomatologia anginosa típica; (3) coronariopatia uniarterial, com lesão passível de tratamento com um único stent; (4) diabéticos ou não diabéticos; (5) doença coronariana estável ou síndrome coronariana aguda.

Os critérios de exclusão adotados foram: (1) doença arterial coronariana (DAC) multiarterial; (2) lesão que necessitasse de abordagem com mais de um stent; (3) angioplastia coronariana prévia com stents; (4) alergia a ácido acetilsalicílico (AAS) e/ou clopidogrel; (5) sangramento intestinal ou geniturinário recente (nos últimos 6 meses); (6) úlcera péptica ativa; (7) cirurgia de grande porte nas últimas 6 semanas; (8) acidente vascular encefálico (AVE) no último ano ou sequela neurológica permanente; (9) gravidez; (10) presença de lesão > 50% no tronco da coronária esquerda.

A seleção dos pacientes se deu de forma sequencial, randômica, na proporção de 1:2, sendo os números sequenciais gerados aleatoriamente por computador (Programa R 2.11). Os pacientes foram, assim, estratificados em dois grupos, de acordo com a indicação para implante de SF ou de SNF.

Grupo SF: 77 pacientes. ICP com implante de SF do tipo zotarolimus (Endeavor Sprint^®^ e Resolute^®^ – Medtronic) em lesões únicas > 70% de estenose, pela estimativa visual da angiografia.

Grupo SNF: 154 pacientes. ICP com implante de SNF em lesões únicas > 70% de estenose, pela estimativa visual da angiografia. Os SNF usados foram: Integrity^®^ (Medtronic), Tsunami^®^ (Terumo) e Tango^®^ (Microport).

A fase hospitalar consistiu em: avaliação das variáveis clínicas; variáveis angiográficas; complicações clínicas; complicações vasculares maiores; eventos cardíacos maiores (morte, oclusão aguda ou subaguda, IAM) e custos.

O seguimento clínico, durante 1 ano, compreendeu a avaliação dos seguintes parâmetros: morte, IAM, angina, reestenose clínica, revascularização da lesão-alvo (RLA), trombose tardia (TT) e custos relacionados com uma nova intervenção, caso houvesse. Os acompanhamentos foram realizados no ambulatório do HUPE e no Hospital São Lucas de Nova Friburgo.

O objetivo da ICP foi sempre obter lesão residual < 10% pela angiografia em cada artéria tratada, sem sinais de dissecção ou trombo que comprometesse o fluxo no vaso em questão. Nos casos de insucesso do procedimento ou necessidade de implante de stent adicional, excluiu-se o paciente do estudo. Durante a intervenção, a administração de qualquer medicação adjuvante ficou a critério do operador. Após a intervenção, os pacientes de ambos os grupos receberam AAS 100 mg/dia e clopidogrel 75 mg/dia, com dupla-antiagregação plaquetária (DAPT) individualizada, variando conforme a prótese empregada, a indicação do médico assistente e o quadro clínico do paciente.

### Análise de Custo-efetividade

**Figura 1:**
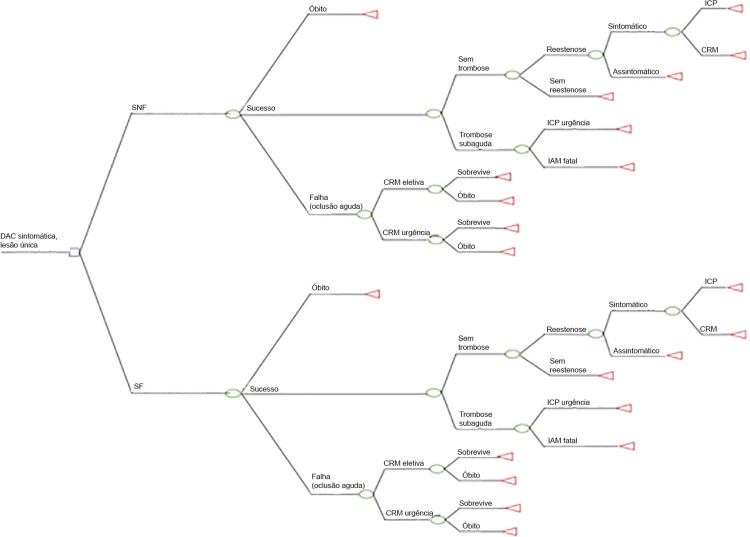
– *Árvore de decisão do SF vs. SNF. SF: stent farmacológico; SNF: stent não farmacológico; CRM: cirurgia de revascularização do miocárdio; DAC: doença arterial coronariana; IAM: infarto agudo do miocárdio; ICP: intervenção coronariana percutânea. Fonte: Polanczyk et al. (2007).^3^*

A população selecionada (pacientes com lesões uniarteriais) foi delineada para um ensaio clínico, considerando as duas alternativas: angioplastia com SF ou angioplastia com SNF. Um modelo analítico foi construído através de uma árvore de decisão ( Figura 1 ) baseada nessas condutas iniciais, numa versão de curto prazo (1 ano), sendo a reestenose clínica evitada o desfecho considerado para o cálculo da efetividade. O modelo utilizou dados probabilísticos de desfechos clínicos oriundos de revisão sistemática de ensaios clínicos randomizados, envolvendo angioplastia coronariana com stent, extraído do estudo de Polanczyk et al.^3^

O custo da angioplastia foi calculado assumindo a perspectiva do SUS, sendo usado como referência os valores reembolsados em internações prévias, com os valores monetários expressos em reais.^3^ O custo do SNF foi considerado a partir do valor reembolsado pelo SUS (R$ 2.034,00). O custo do SF foi considerado a partir do preço médio do mercado do stent eluído com zotarolimus (R$ 3.600,00).

O cálculo da RCEI foi obtido pela diferença de custo entre os dois grupos (internação hospitalar, exames complementares, procedimento percutâneo e preço dos stents) dividida pela diferença de efetividade dos dois grupos (sobrevida livre de reestenose). O valor incremental usado como referência foi o sugerido pela Organização Mundial da Saúde (OMS), de até três vezes o valor do PIB per capita,^11^ que, de acordo com o Instituto Brasileiro de Geografia e Estatística (IBGE), em 2017, era R$ 31.587,00.^12^

### Análise Estatística

Os dados numéricos foram expressos em medidas de tendência central e de dispersão (média, desvio-padrão, mediana e intervalo interquartílico). Os dados categóricos foram expressos em frequências (n) e percentuais (%).

Algumas variáveis numéricas não apresentaram distribuição normal (rejeição da hipótese de normalidade pelo teste de Shapiro-Wilk), sendo aplicado, nesses casos, testes não paramétricos. O critério de determinação de significância adotado foi o nível de 5%. A análise estatística foi processada pelo *software* estatístico SAS System, versão 6.11 (SAS Institute, Inc., Cary, North Carolina).

As variáveis numéricas e categóricas estudadas foram comparadas, considerando-se a utilização do SF e do SNF. Para as variáveis numéricas, utilizou-se o teste *t* de Student (amostras independentes) ou o teste de Mann-Whitney (não paramétrico); para as variáveis categóricas, foi utilizado o teste do qui-quadrado ou exato de Fisher.

A associação das variáveis em estudo com reestenose intrastent (RIS) foi formada pela análise univariada e pela análise multivariada, segundo a regressão logística binária que identificou os preditores independentes pelo método *stepwise forward* . A curva de Kaplan-Meier foi construída para verificar a existência de diferença na sobrevida livre de RIS (clínica) entre os grupos, comparadas pela estatística de *log-rank* . A análise estatística dos custos para elaboração do modelo de decisão foi conduzida pelo programa TreeAge Pro Healthcare (TreeAge Software, Inc., Massachusetts, EUA – versão de 2010). A análise de sensibilidade probabilística multivariável foi realizada com as variáveis de maior impacto no modelo, para testar a robustez do resultado.

## Resultados

Dos 231 participantes do estudo, houve perda de 16 (6,9%) pacientes após a randomização. No grupo SNF (n = 154), 141 (91,5%) pacientes concluíram o seguimento de 1 ano, com registro de 3 (2,1%) óbitos: 2 de origem cardíaca e 1 por AVE. No grupo SF (n = 77), 74 (96,1%) pacientes terminaram o seguimento, com 1 (1,4%) óbito, de origem cardíaca.

A estratificação invasiva durante o acompanhamento foi indicada após aparecimento de angina típica ou após avaliação funcional sugestiva de isquemia. No grupo SNF, 32 (23,2%) pacientes foram estratificados com novo cateterismo: 14 (10,1%) com RIS, 3 com lesões obstrutivas novas e 15 pacientes sem lesões obstrutivas. Dos 14 casos de RIS, 4 foram tratados clinicamente: 1 paciente apresentava reestenose moderada, associada ao aparecimento de nova lesão em outra artéria (tratada com SNF), e 3 pacientes apresentavam lesão difusa e oclusiva, não acometendo a artéria descendente anterior (DA), optando-se por tratamento conservador. Dos 10 casos restantes de RIS, 5 foram tratados com implante de SF, 1 foi tratado com outro SNF, 1 foi submetido a cirurgia de revascularização do miocárdio (CRM) — mamária para descendente anterior — e 3 foram submetidos a angioplastia por balão. Desses 3 pacientes que foram retratados com balão, 1 foi novamente submetido a nova angioplastia no primeiro ano de acompanhamento, com implante de SF.

No grupo SF, 14 (18,9%) pacientes repetiram o cateterismo: 1 com RIS (tratado com outro SF), 1 com lesão nova em outro vaso (tratado com SNF) e 12 sem lesões obstrutivas. Observou-se distribuição semelhante nos grupos, exceto que a angina instável foi mais frequente no grupo SNF (46,5% vs. 30,9%; p = 0,027). No grupo SF, 31,0% pacientes eram diabéticos e, no SNF, 27,7% (p = 0,59), sem significância estatística ( Tabela 1 ).

**Tabela 1:** – Variáveis clínicas e comorbidades dos grupos estudados

Variáveis clínicas	SF		SNF		p-valor
Idade (anos) média ± DP	61,8±10,7		61,9±9,7		0,98*
Sexo masculino n (%)	44	59,5	94	66,7	0,30
Cor branca n (%)	50	73,5	90	67,2	0,35
**Comorbidades n (%)**					
HAS	58	78,4	115	81,6	0,58
Diabetes mellitus	23	31	39	27,7	0,59
Obesidade	18	25,0	31	22,6	0,92
Dislipidemia	43	58,9	75	54,0	0,49
Tabagismo	13	17,8	29	21,2	0,17
História familiar	50	68,5	77	56,6	0,094
IAM prévio	9	12,3	19	13,6	0,80
IRC	3	4,1	4	2,9	0,46
Hemodiálise	1	1,4	1	0,7	0,58
FE < 40%	6	9,0	11	8,5	0,91
Isquemia silenciosa	1	1,4	3	2,2	0,56
Angina estável	23	32,4	46	33,8	0,84
Angina instável	33	46,5	43	30,9	0,027
IAM s/ supra ST	5	6,9	19	14,0	0,13
IAM c/ supra ST	12	16,4	31	22,6	0,29

Os dados categóricos foram expressos em frequência (n) e percentual (%) e comparados pelo teste do qui-quadrado ou exato de Fisher. Os dados numéricos com distribuição normal foram expressos por média ± desvio-padrão e comparados pelo teste t de Student. *para amostras independentes. SF: grupo que recebeu stent farmacológico; SNF: grupo que recebeu stent não farmacológico; DP: desvio-padrão; FE: fração de ejeção; HAS: hipertensão arterial sistêmica; IAM: infarto agudo do miocárdio; IRC: insuficiência renal crônica; supra ST: supradesnivelamento do segmento ST. Fonte: O Autor (2018).

Considerando as variáveis angiográficas, a incidência de lesões tipo C foi 25,4% no grupo SF e 19,9% no SNF, também sem significância estatística. Em ambos os grupos, houve leve predomínio de lesões curtas (< 20 mm): SF com 59,5% e SNF com 54,6% (p = 0,49). Os vasos com calibre < 3,0 mm foram mais frequentes no grupo SF (47,3% vs. 34,0%; p = 0,058) ( Tabelas 2 e 3 ).

**Tabela 2:** – Variáveis angiográficas dos grupos estudados

Variáveis	SF			SNF			p-valor

	n	mediana	Q1-Q3	n	mediana	Q1-Q3	
Diâmetro do stent (mm)**	74	2,95	2,75-3,1	141	3,1	2,75-3,50	0,018**
Comprimento do stent (mm)	74	18,0	15,0-24,0	141	18,0	15,0-26,0	0,97**
**QCA**							
DRV**	46	2,90	2,58-3,19	88	2,89	2,49-3,64	0,56**
% Lesão	45	82,6	72,5-87,9	88	87,1	74,1-93,1	0,069**
Extensão da lesão (mm)	46	7,96	6,37-10,3	86	9,34	6,80-12,7	0,12**
DLM–pré	46	0,805	0,685-1,07	85	0,870	0,610-1,05	0,88**
DLM–pós	46	2,76	2,22-3,26	85	2,86	2,42-3,39	0,32**

Os dados não normais foram expressos por mediana e intervalo interquartílico (Q1-Q3) e comparados pelo teste de Mann-Whitney** (não paramétrico). SF – grupo que recebeu stent farmacológico; SNF: grupo que recebeu stent não farmacológico; DLM: diâmetro luminal mínimo do vaso; DRV; diâmetro de referência do vaso; QCA: Quantitative Coronary Angiography; Q1-Q3: intervalo interquartílico. Fonte: O Autor (2018).

**Tabela 3:** – Variáveis dos procedimentos dos grupos estudados

		SF		SNF		P = Valor
	
		n	%	n	%	
**CASS**	A	4	5,6	5	3,7	0,68
	B1	34	47,9	74	54,4	
	B2	15	21,1	30	22,1	
	C	18	25,4	27	19,9	
**Vaso abordado**	Vaso < 3,0 mm	35	47,3	48	34,0	0,058
	Lesão < 20 mm	44	59,5	77	54,6	0,49
	Coronária direta	12	16,2	48	34,3	0,014
	Circunflexa	4	5,4	13	9,3	
	Descendente anterior	52	70,3	68	48,6	
	Ramos	6	18,2	11	7,8	
**Acesso**	Radial	66	98,5	126	96,97	só descritiva
	Femural	1	1,5	3	2,27	
	Ulnar	0	0	1	0,77	
**Complicação**	Dissecção	0	0	1	0,72	0,67
**Seguimento**	Novo cateterismo	14	18,9	32	23,2	0,47

*Os dados categóricos foram expressos em frequência (n) e percentual (%) e comparados pelo teste do qui-quadrado ou exato de Fisher. SF: grupo que recebeu stent farmacológico; SNF: grupo que recebeu stent não farmacológico; CASS: classificação angiográfica das lesões coronarianas (American Heart Association).Fonte: O Autor (2018).*

Em relação aos desfechos, não existiu diferença estatística em relação a: trombose, infarto, AVE, angina e óbito. Houve mais casos de RIS no grupo SNF (10,1% vs. 1,4%; p=0,018) e, consequentemente, mais casos de RLA (7,3% vs. 1,4%; p=0,058) ( Tabela 4 ).

**Tabela 4:** – Desfechos em um ano de seguimento dos grupos estudados

		SF			SNF		

Desfechos							p-valor
	n		%	n		%	
Sangramento	1		1,4	2		1,4	0,73
RLA	1		1,4	10		7,3	0,058
CRM	0		0	1		0,72	0,66
Angina	16		21,6	39		28,3	0,29
IAM	0		0	1		0,72	0,66
AVE	0		0	3		2,2	0,28
RIS	1		1,4	14		10,1	0,018
Nova lesão	1		1,4	6		4,4	0,23
Óbito	1		1,4	3		2,1	0,58

*Os dados categóricos foram expressos em frequência (n) e percentual (%) e comparados pelo teste do qui-quadrado ou exato de Fisher. SF: grupo que recebeu stent farmacológico; SNF: grupo que recebeu stent não farmacológico; AVE: acidente vascular encefálico; CRM: cirurgia de revascularização do miocárdio; IAM: infarto agudo do miocárdio; RIS: reestenose intrastent; RLA: revascularização da lesão-alvo. Fonte: Autor, 2018.*

**Figura 2:**
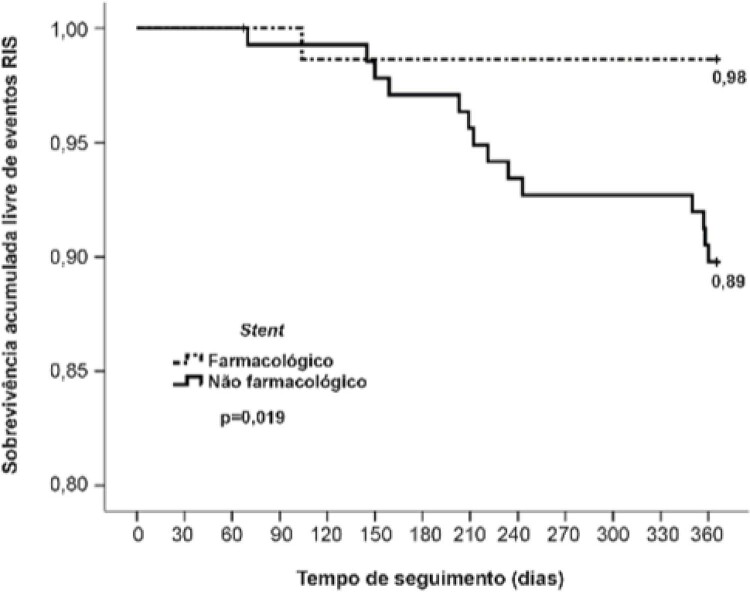
– *Curva de sobrevida livre de evento RIS por tipo de stent. RIS: reestenose intrastent.*

A Figura 2 ilustra a curva de sobrevida livre do evento RIS, pelo método de Kaplan-Meier, no período de acompanhamento em dias, estratificada pelo tipo de stent (SF vs. SNF) e comparada pela estatística *log-rank* . Observou-se que a sobrevida livre de RIS (clínica) no grupo SF foi significativamente maior, com p = 0,019.

### Custo-efetividade

De acordo com o tipo de stent implantado (SF ou SNF), foram calculados os custos do procedimento e a efetividade de cada stent. O SNF apresentou um custo de R$ 4.085,21 e o SF, de R$ 5.722,21. Considerando a RIS, a efetividade do SF foi 8,7% superior em relação ao SNF, com uma RCEI de R$ 18.816,09. Considerando a RLA, a efetividade a favor do SF foi de 5,9%, com RCEI de R$ 27.745,76.

## Discussão

### Análise da População

No presente estudo, como também encontrado na literatura,^13 , 14^ não houve diferença entre o grupo SF e o grupo SNF em relação a eventos adversos maiores (morte, IAM, trombose), com diferença significativa na reestenose (SF: 1,4% e SNF: 10,1%; p = 0,018). O percentual de RLA em um ano foi 1,4% no grupo SF e 7,3% no grupo SNF (p = 0,058). No presente estudo, o único caso de trombose documentado foi no grupo SNF (0,0% vs. 0,74%; p = 0,65), porém sem significado estatístico.

Também em concordância com as diretrizes,^15 , 16^ o uso do acesso radial minimizou os casos de sangramento, não sendo registrado neste trabalho nenhum sangramento maior que justificasse hemotransfusão ou intervenção cirúrgica. Vasos de fino calibre, lesões longas e diabetes mellitus são fatores de risco para reestenose, segundo Singh et al.,^17^ fato não confirmado no presente estudo. Houve uma proporção semelhante de diabéticos nos dois grupos (SF: 31,0% e SNF: 27,7%; p = 0,59). Eram diabéticos 40,0% dos pacientes com RIS; entretanto, 27,7% dos pacientes que não sofreram RIS eram também diabéticos, sem significado estatístico (p = 0,22) ( Tabela 5 ).

**Tabela 5:** – Variáveis clínicas e comorbidades segundo o desfecho RIS

Variáveis clínicas	Com RIS		Sem RIS		p-valor
Idade (anos) média ± DP	59,9±8,4		61,9±10,1		0,45*
Sexo masculino n (%)	9	60,0	127	64,8	0,71
Cor branca n (%)	10	66,7	128	69,6	0,51
**Comorbidades n (%)**					
HAS	13	86,7	156	79,6	0,39
Obesidade	4	26,7	44	23,0	0,74
Diabetes mellitus	6	40	54	27,6	0,22
Dislipidemia	8	53,3	106	54,9	0,91
Tabagismo	3	20,0	38	19,8	0,079
Tabagismo (ex + atual)	13	86,7	116	60,4	0,043
História familiar	9	64,3	116	60,4	0,77
IAM prévio	3	20,0	24	12,4	0,30
IRC	0	0	6	3,1	0,64
Hemodiálise	0	0	2	1,03	0,86
FE < 40%	0	0	16	8,9	0,26
Isquemia silenciosa	1	6,7	3	1,6	0,26
Angina estável	2	13,3	66	35,1	0,086
Angina instável	8	53,3	67	35,1	0,16
IAM s/ supra ST	3	21,4	21	11,1	0,22
IAM c/ supra ST	1	7,1	40	20,8	0,19

Os dados categóricos foram expressos em frequência (n) e percentual (%) e comparados pelo teste do qui-quadrado ou exato de Fisher. Os dados numéricos com distribuição normal foram expressos por média ± desvio-padrão e comparados pelo teste t de Student. *para amostras independentes. RIS: reestenose intrastent; DP: desvio-padrão; FE: fração de ejeção; HAS: hipertensão arterial sistêmica; IAM: infarto agudo do miocárdio; IRC: insuficiência renal crônica; supra ST: supradesnivelamento do segmento ST. Fonte: O Autor (2018).

Em relação à extensão das lesões, em 60,0% dos pacientes com RIS o comprimento da lesão era < 20 mm e 56,6% dos pacientes sem RIS também apresentavam lesões < 20 mm, sem significado estatístico (p = 0,8).

Portanto, o único preditor independente para a reestenose foi o uso do SNF (RR: 8,14; IC95%: 1,05-63,2; p = 0,045), onde 93,3% dos casos de RIS ocorreram em pacientes que usaram o SNF.

### Análise de Custo-efetividade

No Brasil, para o tratamento percutâneo das coronariopatias, é regra o uso do SF no sistema suplementar de saúde, pois esse modelo econômico pauta seu limiar de CE através de demanda, considerando quanto o segurado está disposto a pagar por ele. Entretanto, no SUS, o uso irrestrito do SF ainda é motivo de controvérsias. Por não ter impacto na mortalidade, com diminuição somente no número de reintervenções por reduzir a reestenose, o limiar de CE precisa se basear na oferta, isto é, quanto o Estado está disposto a pagar a mais para obter esse benefício.

O estudo precursor no país para a análise econômica do SF (estudo não randomizado de Polanczyk et al.,^3^ indicou que o custo no primeiro ano do implante de SNF foi R$ 5.788,00 e de SF foi R$ 12.708,00, com efetividade a favor do SF de 13,8%. Utilizando o limiar de CE de USD 10,000.00 por evento evitado, extraído do sistema norte-americano e canadense, concluiu-se que a RCEI do SF de R$ 47.643,00 por reestenose evitada não foi custo-efetiva no SUS.

O presente estudo, randomizado, calculou a RCEI do SF em relação ao SNF, somente no SUS. O custo anual do SF foi R$ 5.722,21 e o custo anual do SNF foi R$ 4.085,21, utilizando o valor de tabela do SUS, que pouco se modificou desde o estudo de Polanczyk et al.,^3^ A efetividade por RIS e por RLA do SF sobre o SNF foi 8,7% e 5,9%, respectivamente, com RCEI de R$ 18.816,09 e de R$ 27.745,76. Com esses resultados, pode-se considerar o SF custo-efetivo?

No Brasil, nunca existiu um valor explícito de limiar de CE como referência para a avaliação da viabilidade econômica de uma tecnologia a ser implementada. A CONITEC^9^ assessora o Ministério da Saúde para a incorporação de qualquer tratamento no SUS, e seus relatórios geralmente utilizam o valor do PIB per capita para estimar esse limiar.^18 - 20^ O uso do PIB per capita como limiar de CE foi recentemente abandonado pela OMS^19^ por falta de especificidade para a tomada de decisões sobre a alocação de recursos. Devido ao cenário de incertezas, existe em tramitação no Senado um projeto de lei que obriga a criação de parâmetros de CE para auxiliar a aprovação de medicamentos, órteses ou próteses no SUS.^21^ Devido à falta de melhor alternativa, o PIB per capita foi o definidor de CE para o presente trabalho.

O preço do SF diminuiu drasticamente. Quando da publicação do trabalho de Polanczyk et al.,^3^ o valor de referência do stent com rapamicina (sirolimus) era R$ 10.320,00 e, no presente estudo, o stent com zotarolimus custou em torno de R$ 3.600,00. Em compensação, o valor do SNF também diminuiu na mesma proporção, apresentando os mesmos avanços tecnológicos da plataforma usada no SF. Curiosamente, o SUS apresenta uma peculiaridade: os valores pagos pelos procedimentos pouco modificaram nos últimos anos, com o preço de sua tabela para o SNF também congelado, porém mais caro atualmente em relação ao do mercado. Apesar da diminuição de seu custo, o último relatório da CONITEC^9^ preconizou o uso do SF no SUS para o grupo de maior risco para reestenose, pagando por ele valor inferior ao pago no mercado.^9^ Seu uso no SUS, portanto, ainda é restrito.

Na Europa, onde o sistema de atenção à saúde é majoritariamente público, o uso do SF já predomina há 5 anos. Em 2013, na França,22 72,5% dos stents implantados eram farmacológicos; no Reino Unido, 89,0%; na Itália, 78,0%; na Alemanha, 77,0% e na Espanha, 74,0%. Barone-Rochette et al.,^23^ em registro de uma coorte de pacientes que implantaram stents revestidos de sirolimus em períodos distintos (2008 e 2012), demonstraram seu CE após a queda de preço. A diferença do custo entre o SES e o SNF era de € 1.200 em 2008 e de € 400 em 2012.

Em 2018, a nova diretriz da *European Society of Cardiology* (ESC) e da *European Association for Cardio-Thoracic Surgery* (EACTS)16 para revascularização do miocárdio recomendou o uso irrestrito do SF, independentemente do tipo de lesão, planejamento de cirurgia não cardíaca ou concomitante anticoagulação. Em suma, o constante aprimoramento do SF e a variedade de modelos oferecidos no mercado tendem a reduzir cada vez mais o seu preço e a massificar o seu uso. O avanço tecnológico deles tende a abolir definitivamente o uso do SNF na prática clínica, faltando somente a mudança de postura dos gestores do governo para implementá-lo de forma mais abrangente, como nos países mais desenvolvidos.

## Conclusões

O SF foi custo-efetivo nos pacientes estudados no SUS comparado ao SNF. Não houve diferença na mortalidade e em outros eventos adversos maiores entre os grupos SF e SNF. O grupo de pacientes que teve implantado o SF apresentou taxa de reestenose clínica significativamente menor em relação ao grupo SNF.

### Limitações do Estudo

Em função da seleção randômica de pacientes uniarteriais sem angioplastia prévia ou história de CRM, houve uma provável seleção de casos de menor complexidade, com menor probabilidade para reestenose, podendo ter influenciado na diferença de efetividade entre os grupos. Além disso, devido ao pequeno número de pacientes estudados, o número de eventos adversos foi reduzido, sendo o uso do SNF o único preditor independente para reestenose, porém com intervalo de confiança muito alargado.
