# Supplementary material for: Custo-efetividade do Stent Farmacológico na Intervenção Coronariana Percutânea no SUS
Source: Arq Bras Cardiol. 2020 Jul 28;115(1):80–9. [Article in Portuguese] doi: 10.36660/abc.20180292 (PMC8384334; doi:10.36660/abc.20180292)
Supplement: Translation

# Translation: Cost-effectiveness of Drug-Eluting Stents in Percutaneous Coronary Intervention in Brazil’s Unified Public Health System (SUS)

**Authors:** João Addison Pessoa, Esmeralci Ferreira, Denizar Viana Araújo, Edirley Maia, Felipe Souza Maia da Silva, Maurício Salles de Oliveira, Denilson Campos de Albuquerque

**Affiliations:** 1 Brazil Universidade do Estado do Rio de Janeiro, Rio de Janeiro, RJ - Brazil; 2 Brazil Hospital São Lucas, Nova Friburgo, RJ - Brazil

**Keywords:** Myocardial Infarction, Percutaneous Coronary Intervention, Drug-Eluting Stents, Coronary Reestenosis, Cost-Benefit Analysis, Unified Health System (SUS

## Abstract

**Background:**

The use of drug-eluting stents (DESs), compared with bare-metal stents (BMSs), in percutaneous coronary intervention (PCI) has reduced the rate of restenosis, without an impact on mortality but with an increase in costs. Medical literature lacks randomized studies that economically compare these 2 stent types within the reality of the Brazilian Unified Public Health System (SUS).

**Objective:**

To estimate the incremental cost-effectiveness ratio (ICER) between DES and BMS in SUS patients with single-vessel coronary artery disease.

**Methods:**

Over a 3-year period, patients with symptomatic single-vessel coronary artery disease were randomized in a 1:2 ratio to receive a DES or BMS during PCI, with a 1-year clinical follow-up. The evaluation included in-stent restenosis (ISR), target lesion revascularization (TLR), major adverse events, and cost-effectiveness for each group. P-values <0.05 were considered significant.

**Results:**

In the DES group, of 74 patients (96.1%) who completed the follow-up, 1 developed ISR (1.4%), 1 had TLR (1.4%), and 1 died (1.4%), with no cases of thrombosis. In the BMS group, of 141 patients (91.5%), ISR occurred in 14 (10.1%), TLR in 10 (7.3%), death in 3 (2.1%), and thrombosis in 1 (0.74%). In the economic analysis, the cost of the procedure was R$ 5,722.21 in the DES group and R$ 4,085.21 in the BMS group. The effectiveness by ISR and TLR was 8.7% for DES and 5.9% for BMS, with an ICER of R$ 18,816.09 and R$ 27,745.76, respectively.

**Conclusions:**

In the SUS, DESs were cost-effective in accordance with the cost-effectiveness threshold recommended by the World Health Organization (Arq Bras Cardiol. 2020; 115(1):80-89)

## Introduction

Data extracted from the 2013 Brazilian National Health Interview Survey^1^ estimated that 72.1% of the population would use the Unified Public Health System (SUS) for medical or dental treatment. According to the number of deaths per cause between 2004 and 2014 in Brazil, it was estimated that 1,069,653 (8.8%) individuals died from acute myocardial infarction (AMI) or other ischemic heart diseases. In this respect, it is important to develop sustainable measures for the prevention and treatment of these illnesses in the SUS.^2^

In Brazil, the first drug-eluting stents (DESs) were restricted to the supplementary health system due to their high cost. Initial studies, conducted both in Brazil and abroad, have not demonstrated cost-effectiveness for DESs in all cases, suggesting their use in situations of greater risk for restenosis.^3 - 6^

The limitations described above led to the development of new DESs, called second-generation DESs. With new antiproliferative drugs and improved platform with thinner metal struts (chromium-cobalt, platinum-cobalt alloys), they provided better stent apposition and less contact area for endothelialization. Biocompatible polymers reduced the local inflammatory process, reducing the occurrence of late thrombosis.^7 , 8^

More than a decade after the beginning of their marketing, the use of DES in the SUS remained limited despite lower cost and more favorable results. In 2014, the Brazilian National Committee for Health Technology Incorporation (CONITEC)^9^ recognized the cost-benefit of DES implantation in patients with diabetes, small vessels (<2.5 mm), and long lesions (>18 mm). Although the market price of DESs is higher than that of bare-metal stents (BMSs), the price suggested in the SUS (R$ 2,034.50 / code 070204061-4) was the same for both stent types, hindering the systematic use of DESs in private services involved in agreements with the Brazilian Ministry of Health.

According to data from the DATASUS,^10^ in 2008, 44,138 percutaneous coronary interventions (PCIs) were performed with or without stenting. Eight years later, 79,997 PCIs were performed. With this significant increase in procedures (72.84%), it is possible to project an increase in cases of restenosis that could potentially be reduced with more liberal use of DESs in the SUS.

Despite the favorable scenario for the full incorporation of DESs into the SUS, scientific evidence based on the Brazilian reality is lacking. Therefore, this study aimed to analyze and estimate the incremental cost-effectiveness ratio (ICER) between DESs and BMSs in SUS patients.

## Objectives

To evaluate the cost-effectiveness and major adverse events of DESs compared with BMSs in patients with single-vessel coronary artery disease undergoing PCI.

## Methods

We conducted a randomized clinical study of patients undergoing PCI from November 2013 to October 2016 at Hospital Universitário Pedro Ernesto (HUPE) and at Hospital São Lucas de Nova Friburgo (HSL), Brazil. The study was approved by the Research Ethics Committees of both institutions, under number 923660. Written informed consent was obtained from each study participant, in accordance with the Brazilian National Health Council Resolution No. 466/2012.

A total of 231 patients of both sexes with single-vessel lesions, an indication for PCI after preliminary coronary cineangiography, and symptoms of angina or noninvasive tests showing myocardial ischemia were assessed. The inclusion criteria were (1) age ≥18 years, (2) angiographically significant lesions (>70%) in a coronary artery of great anatomical importance, with irrigation of a large area of cardiac muscle, related to the presence of ischemia or typical angina symptoms, (3) single-vessel coronary artery disease, with a lesion amenable to treatment with a single stent, (4) presence or not of diabetes, and (5) stable coronary disease or acute coronary syndrome. The exclusion criteria were (1) multivessel coronary artery disease, (2) injury that required an approach with more than one stent, (3) previous coronary angioplasty with stenting, (4) allergy to aspirin and/or clopidogrel, (5) recent intestinal or genitourinary bleeding (in the last 6 months), (6) active peptic ulcer, (7) major surgery in the past 6 weeks, (8) stroke in the last year or permanent neurological sequelae, (9) pregnancy, and (10) presence of lesion >50% in the left main coronary artery.

Participants were recruited sequentially and randomly assigned in a 1:2 ratio to receive a DES or BMS, according to a computer-generated list of random numbers (Program R 2.11). The DES group consisted of 77 patients who underwent PCI with implantation of a zotarolimus-eluting stent (Endeavor Sprint and Resolute, Medtronic) in single lesions with stenosis of >70% by visual estimation on angiography. The BMS group consisted of 154 patients who underwent PCI with implantation of a BMS in single lesions with stenosis of >70% by visual estimation on angiography. The BMSs used were Integrity (Medtronic), Tsunami (Terumo), and Tango (Microport).

In-hospital evaluation included the assessment of clinical variables, angiographic variables, clinical complications, major vascular complications, major cardiac events (death, acute or subacute occlusion, and AMI), and costs. The 1-year clinical follow-up included the assessment of the following parameters: death, AMI, angina, in-stent restenosis (ISR), target lesion revascularization (TLR), late thrombosis, and costs related to re-intervention, if any. Follow-ups were conducted at the HUPE outpatient clinic and at HSL.

The aim of PCI was always to obtain a residual lesion <10% on angiography in each treated artery, without signs of dissection or thrombus that would compromise the flow of the vessel. Patients in whom the procedure failed or who required additional stent implantation were excluded from the study. During intervention, any adjuvant medication was administered at the physician’s discretion. After intervention, patients in both groups received dual antiplatelet therapy (DAPT) with aspirin (100 mg/day) and clopidogrel (75 mg/day), tailoring the duration of DAPT according to the type of stent used, the indication of the physician, and the clinical condition of the patient.

### Cost-effectiveness Analysis

The study population was selected for a clinical trial considering 2 alternatives: PCI with DES or PCI with BMS. An analytical model was constructed by using a decision tree ( Figure 1 ) based on these initial procedures, in a short-term version (1 year). Each avoided ISR was considered for the calculation of effectiveness. The model used probabilistic data from clinical outcomes of a systematic review of randomized clinical trials involving coronary angioplasty with stenting, extracted from the study by Polanczyk et al.^3^

**Figure 1:** – *CABG:coronary artery by-pass graft; PCI-BMS:percutaneous coronary intervention witn bare metal stent; PCI-DES: percutaneous coronary intervention with drug elution stent. Source: Polanczyk et al(2007)^3^*

The cost of angioplasty was calculated from the perspective of the SUS, using as a reference the amounts reimbursed for previous hospitalizations, with monetary values expressed in Brazilian currency (R$).^3^ The cost of BMS was defined as the amount reimbursed by the SUS (R$ 2,034.00). The cost of DES was defined as the average market price of zotarolimus-eluting stents (R$ 3,600.00).

ICER was calculated by dividing the difference in costs (hospitalization, complementary tests, percutaneous procedures, and stent price) between the 2 groups by the difference in effectiveness (restenosis-free survival) between the 2 groups. The incremental value suggested by the World Health Organization (WHO) was used as a reference: up to 3 times the value of the GDP per capita,^11^ which, according to the Brazilian Institute of Geography and Statistics (IBGE), was R$ 31,587.00 in 2017.^12^

### Statistical Analysis

Numerical data were expressed as measures of central tendency and dispersion (mean, standard deviation, median, and interquartile range). Categorical data were expressed as frequencies (n) and percentages (%). Numerical variables with non-normal distribution (normality hypothesis rejected by the Shapiro-Wilk test) were analyzed using nonparametric tests. Numerical and categorical variables were compared considering the use of DES or BMS. Student’s *t* test for independent samples or the Mann-Whitney (nonparametric) test were used for the numerical variables, whereas the chi-square test or Fisher’s exact test were used for the categorical variables. The statistical analysis was performed using SAS System, version 6.11 (SAS Institute, Inc, Cary, North Carolina). The level of significance was set at 5% for all analyses.

The association of the variables under study with ISR was determined by univariate and multivariate analyses, according to the independent predictors identified by the forward stepwise binary logistic regression analysis. A Kaplan-Meier curve was used to analyze differences in ISR-free survival between the 2 groups, which were compared by the log-rank test.

A decision tree model using TreeAge Pro Healthcare, version 2010 (TreeAge Software, Inc., Williamstown, MA, USA), was developed for cost analysis. A multivariable probabilistic sensitivity analysis was conducted with the variables with the greatest impact on the model in order to test the robustness of the result.

## Results

Of 231 patients initially included in the study, 16 (6.9%) were lost after randomization. In the BMS group (n=154), 141 (91.5%) patients completed 1 year of follow-up, with 3 (2.1%) deaths: 2 from cardiac causes and 1 from stroke. In the DES group (n=77), 74 (96.1%) patients completed 1 year of follow-up, with 1 (1.4%) death from cardiac causes.

During follow-up, invasive stratification was indicated after the onset of typical angina or after functional assessment suggestive of ischemia. In the BMS group, 32 (23.2%) patients were stratified with a second catheterization: 14 (10.1%) with ISR, 3 with new obstructive lesions, and 15 without obstructive lesions. Of 14 ISR cases, 4 were treated clinically: 1 patient had moderate restenosis associated with the development of a new lesion in another artery (treated with BMS implantation), and 3 patients had a diffuse, occlusive lesion that did not affect the anterior descending artery and were treated conservatively. Of the 10 remaining ISR cases, 5 were treated with DES implantation, 1 was treated with implantation of another BMS, 1 underwent coronary artery bypass grafting (CABG), and 3 underwent balloon angioplasty. Of the 3 patients treated with balloon angioplasty, 1 underwent a second PCI with DES implantation. In the DES group, 14 (18.9%) patients repeated catheterization: 1 with ISR (treated with implantation of another DES), 1 with a new lesion in another vessel (treated with BMS implantation), and 12 without obstructive lesions.

A similar distribution was observed for the 2 groups, except for more frequent unstable angina in the BMS group (46.5% vs 30.9%; p = 0.027). In the DES group, 31.0% of patients had diabetes, against 27.7% in the BMS group (p = 0.59), without statistically significant difference between the groups ( Table 1 ).

**Table 1:** Clinical variables and comorbidities of the study groups

Clinical variables	DES		BMS		p-value
Mean age (years) ± SD	61.8 ± 10.7		61.9 ± 9.7		0.98 *
Male sex n (%)	44	59.5	94	66.7	0.30
White color n (%)	50	73.5	90	67.2	0.35
**Comorbidities n (%)**					
Hypertension	58	78.4	115	81.6	0.58
Diabetes mellitus	23	31	39	27.7	0.59
Obesity	18	25.0	31	22.6	0.92
Dyslipidemia	43	58.9	75	54.0	0.49
Smoking	13	17.8	29	21.2	0.17
Family history	50	68.5	77	56.6	0.094
Previous AMI	9	12.3	19	13.6	0.80
CRF	3	4.1	4	2.9	0.46
Hemodialysis	1	1.4	1	0.7	0.58
EF <40%	6	9.0	11	8.5	0.91
Silent ischemia	1	1.4	3	2.2	0.56
Stable angina	23	32.4	46	33.8	0.84
Unstable angina	33	46.5	43	30.9	0.027
NSTEMI	5	6.9	19	14.0	0.13
STEMI	12	16.4	31	22.6	0.29

Categorical data were expressed as frequency (n) and percentage (%) and compared by the X^2^test or Fisher's exact test. Numerical data with normal distribution were expressed as mean ± standard deviation and compared by Student's t test * for independent samples. Legend: DDES - drug-eluting stent; BMS - bare-metal stent; SD - standard deviation; EF - ejection fraction; AMI - acute myocardial infarction; CRF - chronic renal failure; STEMI: ST-segment elevation myocardial infarction; NSTEMI: non-ST-segment elevation myocardial infarction. Source: The Author, 2018.

Regarding angiographic variables, the rate of type C lesions was 25.4% in the DES group and 19.9% in the BMS group, with no between-group difference. In both groups, there was a slight predominance of short lesions (<20 mm): 59.5% in the DES group and 54.6% in the BMS group (p = 0.49). Vessels with a diameter of <3.0 mm were more frequent in the DES group (47.3% vs 34.0%; p = 0.058) ( Tables 2 and 3 ) .

**Table 2:** Angiographic variables of the study groups

Variables	DES			BMS			p-value

	n	median	Q1-Q3	n	median	Q1-Q3	
Stent diameter (mm) **	74	2.95	2.75 - 3.1	141	3.1	2.75 - 3.50	0.018 **
Stent length (mm)	74	18.0	15.0 - 24.0	141	18.0	15.0 - 26.0	0.97 **
QCA							
RDV **	46	2.90	2.58 - 3.19	88	2.89	2.49 - 3.64	0.56 **
% Lesion	45	82.6	72.5 - 87.9	88	87.1	74.1 - 93.1	0.069 **
Lesion extension (mm)	46	7.96	6.37 - 10.3	86	9.34	6.80 - 12.7	0.12 **
MLD – pre	46	0.805	0.685 - 1.07	85	0.870	0.610 - 1.05	0.88 **
MLD – post	46	2.76	2.22 - 3.26	85	2.86	2.42 - 3.39	0.32 **

Data with non-normal distribution were expressed as median and interquartile range (Q1-Q3) and compared by the Mann-Whitney ** (nonparametric) test. DES - drug-eluting stent; BMS - bare-metal stent; MLD - minimal lumen diameter; RDV - reference diameter of the vessel; QCA - quantitative coronary angiography; Q1-Q3 - interquartile range. Source: The Author, 2018.

**Table 3:** – Procedure-related variables of the study groups

		DES		BMS		p value

		n	%	n	%	
**CASS**	A	4	5.6	5	3.7	0,68
	B1	34	47.9	74	54.4	
	B2	15	21.1	30	22.1	
	C	18	25.4	27	19.9	
**Treated vessel**	Vessel <3.0 mm	35	47.3	48	34.0	0,058
	Lesion <20 mm	44	59.5	77	54.6	0,49
	Right coronary artery	12	16.2	48	34.3	0,014
	Circumflex artery	4	5.4	13	9.3	
	LADA	52	70.3	68	48.6	
	Branch	6	18.2	11	7.8	
**Access**	Radial	66	98.5	126	96.97	descriptive only
	Femoral	1	1.5	3	2.27	
	Ulnar	0	0	1	0.77	
**Complication**	Dissection	0	0	1	0.72	0,67
**Follow-up**	New coronarography	14	18.9	32	23.2	0,47

*Categorical data were expressed as frequency (n) and percentage (%) and compared using the X^2^test or Fisher’s exact test. DES: drug-eluting stent; BMS: bare-metal stent; ACCL: angiographic classification of coronary lesions (American Heart Association); LAD: left anterior descending artery. Source: The Author, 2018.*

The 2 groups did not differ in the occurrence of thrombosis, infarction, stroke, angina, or death. The BMS group had more cases of ISR (10.1% vs 1.4%; p = 0.018) and, consequently, more cases of TLR (7.3% vs. 1.4%; p = 0.058) ( Table 4 ).

**Table 4:** – Outcomes at 1-year follow-up for the study groups

Outcomes	DES		BMS		p-value

	n	%	n	%	
Bleeding	1	1.4	2	1.4	0.73
TLR	1	1.4	10	7.3	0.058
CABG	0	0	1	0.72	0.66
Angina	16	21.6	39	28.3	0.29
AMI	0	0	1	0.72	0.66
Stroke	0	0	3	2.2	0.28
ISR	1	1.4	14	10.1	0.018
New injury	1	1.4	6	4.4	0.23
Death	1	1.4	3	2.1	0.58

*Categorical data were expressed as frequency (n) and percentage (%) and compared using the x 2 test or Fisher’s exact test. DES: drug-eluting stent; BMS: bare-metal stent; CABG: coronary artery bypass graft; AMI - acute myocardial infarction; CRF - chronic renal failure; ISR - in-stent restenosis; TLR - target lesion revascularization . Source: Author, 2018.*

Figure 2 shows the Kaplan-Meier restenosis-free survival curve during follow-up (in days), stratified by stent type (DES × BMS) and compared by the log-rank test. Restenosis-free survival was significantly higher in the DES group than in the MBS group (p = 0.019).

**Figure 2:** – *Event-free survival curve by stent type.*

### Cost-effectiveness Analysis

The costs of the procedure and the effectiveness of each stent were calculated according to the type of stent implanted (DES or BMS). BMS had a cost of R$ 4,085.21 and DES of R$ 5,722.21. Considering the occurrence of ISR, DESs were 8.7% more effective than BMSs, with an ICER of R$ 18,816.09. Regarding TLR, DESs were 5.9% more effective than BMSs, with an ICER of R$ 27,745.76.

## Discussion

### Population Analysis

In the present study, as previously reported in the literature,^13 , 14^ there was no difference between the use of DESs and BMSs in major adverse events (death, AMI, thrombosis), but a significant difference was observed in restenosis (DES: 1.4 % vs BMS: 10.1%; p = 0.018). The rate of TLR in 1 year was 1.4% in the DES group and 7.3% in the BMS group (p = 0.058). In this study, the only documented case of thrombosis occurred in the DES group (0.0% vs. 0.74%; p = 0.65), but without statistical significance.

In accordance with national and international guidelines for PCI,^15 , 1,6^ the use of radial access minimized the occurrence of bleeding, with no major bleeding requiring blood transfusion or surgical intervention. Although small-diameter vessels, long lesions and diabetes mellitus are risk factors for restenosis,^17^ this was not confirmed in the present study. Presence of diabetes did not differ between the 2 groups (DES: 31.0% and BMS: 27.7%; p = 0.59). Of patients with ISR, 40.0% had diabetes; however, 27.7% of patients who did not develop ISR also had diabetes, with no statistical significance (p = 0.22) ( Table 5 ). Regarding lesion length, lesions <20 mm were found in 60.0% of patients with ISR and in 56.6% of patients without ISR, without statistical significance (p = 0.8).

**Table 5:** Clinical variables and comorbidities according to the ISR outcome

Clinical variables	WITH ISR		WITHOUT ISR		p-value
Mean age (years) ± SD	59.9 ± 8.4		61.9 ± 10.1		0.45 *
Male sex n (%)	9	60.0	127	64.8	0.71
White color n (%)	10	66.7	128	69.6	0.51
**Comorbidities n (%)**					
Hypertension	13	86.7	156	79.6	0.39
Obesity	4	26.7	44	23.0	0.74
Diabetes mellitus	6	40	54	27.6	0.22
Dyslipidemia	8	53.3	106	54.9	0.91
Smoking	3	20.0	38	19.8	0.079
Smoking (ex + current)	13	86.7	116	60.4	0.043
Family history	9	64.3	116	60.4	0.77
Previous AMI	3	20.0	24	12.4	0.30
CRF	0	0	6	3.1	0.64
Hemodialysis	0	0	2	1.03	0.86
EF <40%	0	0	16	8.9	0.26
Silent ischemia	1	6.7	3	1.6	0.26
Stable angina	2	13.3	66	35.1	0.086
Unstable angina	8	53.3	67	35.1	0.16
NSTEMI	3	21.4	21	11.1	0.22
STEMI	1	7.1	40	20.8	0.19

Categorical data were expressed as frequency (n) and percentage (%) and compared by the X^2^test or Fisher’s exact test. Data with normal distribution were expressed as mean ± standard deviation and compared by Student's t test * for independent samples. Legend: ISR - in-stent restenosis; SD - standard deviation; EF - ejection fraction; AMI - acute myocardial infarction; CRF - chronic renal failure; STEMI: ST-segment elevation myocardial infarction; NSTEMI: non-ST-segment elevation myocardial infarction. Source: The Author, 2018.

Therefore, the only independent predictor of restenosis was the use of a BMS (RR: 8.14; 95% CI: 1.05-63.2; p = 0.045), where 93.3% of ISR cases occurred in patients who received a BMS.

### Cost-effectiveness Analysis

In Brazil, the use of DESs for PCI is a rule in the supplementary health system, as this economic model bases its cost-effectiveness threshold on demand, considering how much the insured is willing to pay for it. However, the unrestricted use of DESs in the SUS is still a matter of controversy. As their use does not have an impact on mortality, with a decrease only in the number of re-interventions due to a reduction in restenosis, the cost-effectiveness threshold needs to be based on supply, that is, on how much more the State is willing to pay to obtain such a benefit.

Polanczyk et al.,^3^ in a previous non-randomized study conducted in Brazil for the economic analysis of DESs, reported that the cost in the first year of implantation was R$ 5,788.00 for BMSs and R$ 12,708.00 for DESs, with a 13.8% higher effectiveness in favor of DESs. Using a cost-effectiveness threshold of USD 10,000.00 per avoided event, extracted from the North American and Canadian systems, it was concluded that the ICER of R$ 47,643.00 for DESs per avoided restenosis was not cost-effective in the SUS.

The present randomized study calculated the ICER of DESs in relation to BMSs only in the SUS. According to the SUS’s reference values, the annual cost of DESs was R$ 5,722.21 and the annual cost of BMSs was R$ 4,085.21, which has changed little since the study by Polanczyk et al.^3^ The effectiveness by ISR and TLR was 8.7% for DESs and 5.9% for BMSs, with an ICER of R$ 18,816.09 and R$ 27,745.76, respectively. Based on these results, can we consider DESs cost-effective?

In Brazil, there has never been an explicit threshold value for cost-effectiveness as a reference for assessing the economic viability of a technology to be implemented. CONITEC,^9^ an adviser to the Ministry of Health for the incorporation of any treatment into the SUS, often uses the value of the GDP per capita in its reports to estimate this threshold.^18 - 20^ The use of the GDP per capita as a threshold for cost-effectiveness has recently been abandoned by the WHO^19^ due to lack of specificity for decision-making on resource allocation. Because of a scenario of uncertainty, there is a bill in the Senate that proposes the creation of cost-effectiveness parameters to assist in the approval of drugs, orthoses, and prostheses in the SUS.^21^ In the absence of a better alternative, the GDP per capita was the parameter used to define cost-effectiveness in the present study.

The DES price has dropped dramatically. At the time of the study by Polanczyk et al.,^3^ the reference price of rapamycin-eluting stents was R$ 10,320.00, whereas, in the present study, the price of zotarolimus-eluting stents was around R$ 3,600.00. The price of BMSs decreased as well, while incorporating the same technological advances of the platform used in DESs. Interestingly, SUS has a peculiarity: the amounts paid for the procedures have changed little over recent years, where, although the price of DESs in the SUS remained unchanged, they are currently more expensive in relation to the market price. Despite the decrease in their cost, the latest CONITEC report^9^ recommended the use of DESs in the SUS only for patients at greatest risk for restenosis, purchasing them at a price below the market price.^9^ Their use in the SUS, therefore, is still restricted.

In Europe, where the health care system is mostly public, DESs have been widely used for 5 years. In 2013, in France,^22^ 72.5% of implanted stents were DESs; in the United Kingdom, 89.0%; in Italy, 78.0%; in Germany, 77.0%; and in Spain, 74.0%. Barone-Rochette et al.,^23^in a cohort study of patients who received sirolimus-eluting stents at different time points (2008 and 2012), demonstrated their cost-effectiveness after the price drop. The cost difference between DESs and BMSs was € 1200 in 2008 and € 400 in 2012.

In 2018, the new European Society of Cardiology (ESC)/European Association for Cardio-Thoracic Surgery (EACTS)^16^ guideline for myocardial revascularization recommended the unrestricted use of DESs, regardless of the type of injury, planning for non-cardiac surgery, or concomitant anticoagulation. In short, the constant improvement of DESs and the variety of models available on the market tend to further reduce their price and increase their use. Technological advances in DESs tend to ultimately eliminate the use of BMSs in clinical practice, but a change of attitude of government managers is still lacking to implement their use more broadly, as in developed countries.

## Conclusions

DESs were cost-effective in the SUS patients participating in the study, compared with BMSs. There was no difference in mortality or other major adverse events between DESs and BMSs. Patients who received a DES had a significantly lower rate of ISR compared with those who received a BMS.

### Study Limitations

Due to the random selection of patients with single-vessel coronary artery disease without previous angioplasty or history of CABG, less complex cases were probably selected, with a lower probability of developing restenosis, which may have influenced the difference in effectiveness between the groups. In addition, due to the small sample size, the number of adverse events was low, and the use of BMSs was the only independent predictor of restenosis, but with a wide confidence interval.
